# SEOM-GEINO clinical guidelines for high-grade gliomas of adulthood (2022)

**DOI:** 10.1007/s12094-023-03245-y

**Published:** 2023-08-04

**Authors:** Pedro Pérez Segura, Noelia Vilariño Quintela, María Martínez García, Sonia del Barco Berrón, Regina Gironés Sarrió, Jesús García Gómez, Almudena García Castaño, Luis Miguel Navarro Martín, Oscar Gallego Rubio, Estela Pineda Losada

**Affiliations:** 1 Medical Oncology Department, Hospital Clínico San Carlos, IdISCC, Madrid, Spain; 2 Medical Oncology Department, Catalan Institute of Oncology, Barcelona, Spain; 3 Preclinical and Experimental Research in Thoracic Tumors (PReTT) Group, Oncobell Program, IDIBELL, L’Hospitalet, Barcelona, Spain; 4 Medical Oncology Department, Hospital del Mar, Barcelona, Spain; 5 Cancer Research Program, Hospital del Mar Research Institute, Barcelona, Spain; 6 Medical Oncology Department, Unidad Cáncer de Mama y Tumores Cerebrales, Instituto Catalán de Oncologia, Hospital Universitario Doctor Josep Trueta, Girona, Spain; 7 Medical Oncology Department. Hospital, Univeristari i Politècnic La Fe, Valencia, Spain; 8 Medical Oncology Department, Complejo Hospitalario Universitario de Orense, Orense, Spain; 9 Medical Oncology Department, Hospital Universitario Marqués de Valdecilla, Santander, Spain; 10 Medical Oncology Department, Complejo Asistencial Universitario de Salamanca, Salamanca, Spain; 11 Medical Oncology Department, Hospital de Sant Pau i La Santa Creu, Barcelona, Spain; 12 Medical Oncology Department, Hospital Clinic and Translational Genomics and Targeted Therapies in Solid Tumors, IDIBAPS, Barcelona, Spain

**Keywords:** High-grade gliomas, Management, Epidemiology, Molecular, Treatment

## Abstract

High-grade gliomas (HGG) are the most common primary brain malignancies and account for more than half of all malignant primary brain tumors. The new 2021 WHO classification divides adult HGG into four subtypes: grade 3 oligodendroglioma (1p/19 codeleted, IDH-mutant); grade 3 IDH-mutant astrocytoma; grade 4 IDH-mutant astrocytoma, and grade 4 IDH wild-type glioblastoma (GB). Radiotherapy (RT) and chemotherapy (CTX) are the current standard of care for patients with newly diagnosed HGG. Several clinically relevant molecular markers that assist in diagnosis and prognosis have recently been identified. The treatment for recurrent high-grade gliomas is not well defined and decision-making is usually based on prior strategies, as well as several clinical and radiological factors. Whereas the prognosis for GB is grim (5-year survival rate of 5–10%) outcomes for the other high-grade gliomas are typically better, depending on the molecular features of the tumor. The presence of neurological deficits and seizures can significantly impact quality of life.

## Incidence and epidemiology

GB is one of the most aggressive malignancies, as well as the most common malignant primary tumor of the brain, accounting for 14.5% of all central nervous system (CNS) tumors and 48.6% of malignant brain tumors [1]. The median overall survival (OS) of GB patients is 15 months [1, 2].

The incidence of primary brain tumors has been increasing over recent decades, especially in older adults, and the incidence of GB varies, depending on the report, from 3.19 to 4.17 case per 100,000 person-years [3, 4]. Ostrom et al. [4] presented an age-adjusted incidence rate of 0.18 (95% CI 0.16–0.19) per 100,000 people in the 0–19 year-old age group.

Elderly people represent a consistent population of GB patients. According to the CBTRUS (Central Brain Tumor Registry of the United States) statistical report covering the 2013–2017 period, the incidence of GB is 3.23 per 100,000 people per year and is higher among people over the age of 40 years (6.97 per 100,000 people per year) and reaching its peak in 75–84 year olds (15.30 per 100,000 people per year) [4].

Glioblastoma multiforme location is predominantly concentrated in the frontal, temporal, and parietal lobes, and less often, it affects other structures. In the last 2 decades, the increase in the number of cases detected (increased morbidity/better diagnostic techniques) has been especially striking, particularly in the frontal and temporal lobes [5].

All studies presented indicate a higher incidence of GB in men, 1.6 times more [6]. Rare hereditary syndromes, such as neurofibromatosis type 1 and Cowden, Turcot, Lynch, and Li-Fraumeni syndromes confer an increased risk for glioma.

Age significantly affects the incidence of GB, in that the vast majority of cases occur in people over 40 years of age. In 47.9% of the subjects, the age at the time of GB diagnosis was > 65 years; similarly, 46.3% of the subjects were between 40 and 64 years of age [4].

There is a limited association between specific ethnic groups and the risk of developing GB. Bohn et al. [7] reported a 2.97 times higher incidence of GB in Caucasians compared to Asians, and a 1.99 times higher incidence in Caucasians compared to African Americans.

A literature review by Bowers et al. [8] in 2013 documented an 8.1–52.3 times increased risk of CNS cancer after RT to the head for a CNS tumor in childhood. A meta-analysis conducted by Ahn et al. [9] reported an increased risk of malignant brain tumors associated with lead exposure (pooled OR = 1.13, 95% CI: 1.04–1.24).

With the popularization of electronic devices, such as microwave ovens and cell phones, the impact of exposure to electromagnetic waves and the risk of developing CNS tumors became a controversial topic. Today, people are commonly exposed to radio-frequency electromagnetic fields (RF-EMF) through electronic devices, such as cell phones, cordless phones, radios, and Bluetooth. Olson et al. concluded that, despite the high risk of error in the studies available, the potential carcinogenic effects of RF-EMF cannot be ruled out [10].

High-grade IDH-mutant astrocytomas (grades 3 and 4) are uncommon in adults. In Europe, the annual incidence of grade 3 astrocytomas is approximately 0.3 per 100,000. In population-based registries, they constitute 4% of all malignant tumors of the CNS [11, 12]. IDH-mutant astrocytomas typically occur in younger patients, often in their fourth and fifth decades of life. Grade 3 oligodendrogliomas are relatively rare and have much better prognosis compared to other HGG.

## Methodology

This guideline is based on a systematic review of relevant published studies and with the consensus of ten oncologists with great expertise in treatment from GEINO (Spanish Group of Investigation in Neuro-Oncology) and SEOM (Spanish Society of Medical Oncology), as well as an external review panel consisting of two experts designated by SEOM. The Infectious Diseases Society of America-US Public Health Service Grading System for Ranking Recommendations in Clinical Guidelines has been used to assign levels of evidence and grades of recommendation.

### Diagnosis, pathology, and molecular diagnosis

The current diagnostic process is based on the 5th edition of the WHO’s classification (2021) [13] and the recommendations of cIMPACT-NOW [14–16], integrating a histological and molecular classification. There are several considerations to bear in mind regarding the differences between the 2016 and 2021 editions: adult and pediatric gliomas have been separated; the grading system is expressed using arabic numerals; the term “anaplastic” has been deleted, and the nomenclature NOS (not otherwise specified) and NEC (not elsewhere specified) have been introduced. NOS is used when the diagnostic tests necessary to reach to a specific WHO diagnosis cannot be performed or have failed and NEC when the necessary analyses are performed, but the results do not establish a specific entity.

Based on this, the following biomarkers are critical for categorizing adult gliomas: IDH mutation, 1p/19q codeletion, histone H3 K27M alterations, histone H3.3 G34R/V mutation, TERT promoter mutation, EGFR gene amplification, chromosome 7 combined with loss of chromosome 10 (+ 7/–10), and homozygous deletions at 9p21 involving the CDKN2A and CDKN2B gene loci.

Diffuse gliomas that are immunohistochemically negative for IDH1 R132H should be sequenced for the less common IDH1 and IDH2 mutations, except in patients over the age of 55 years. IDH-mutant astrocytomas generally also have a loss of ATRX nuclear expression and P53 mutations, but by definition, lack 1p/19q deletions [13]. Nuclear ATRX positivity (ATRX wild-type) in an IDH-mutant glioma should prompt 1p/19q codeletion analysis to distinguish between an IDH-mutant astrocytoma and an oligodendroglioma. Oligodendroglial tumors are defined as IDH-mutant gliomas with the presence of the 1p/19q codeletion [13]. Astrocytic gliomas with wild-type IDH, necrosis, and/or microvascular proliferation are classified as WHO grade 4 IDH wild-type glioblastomas (GB). The presence of (+ 7/-10), EGFR amplification, and TERT promoter mutation are diagnostic of grade 4 GB in all IDH wild-type (WT) gliomas, even in the absence of necrosis, proliferation, or microvascularization. If one or more of them are present, these tumors are classified as grade 4 IDH wild-type GB [17]. WHO grade 4 H3 K27M-altered diffuse midline gliomas are defined as a diffuse glioma located in midline structures, such as the thalamus, pons, brainstem, and spinal cord. Hemispheric glioma has been proposed as a new subtype of malignant glioma, characterized by missense mutations affecting codon 34 of H3.3 G3A4. Gliomas with histone 3 alterations (H3 K27M and H3.3 G34A4) belong to pediatric HGG, but also occur in the young adult population; thus, it is important to include histone 3 alterations in the adult diagnostic algorithm of HGG. MGMT promoter methylation is of limited diagnostic value, but might inform treatment decisions [18]. The methylation status of the MGMT promoter should be analyzed by methylation-specific PCR, MLPA or pyrosequencing [19]. Homozygous CDKN2A/B deletion is indicative of poor prognosis and a marker of WHO grade 4 in all IDH-mutant astrocytomas. Next-generation sequencing-based gene panels might enable all or most relevant genetic and chromosomal aberrations to be evaluated with a single assay [20]. Recently, methylome profiling has emerged as a powerful approach to brain tumors classification, but technology is currently not widely available [13].

#### Algorithm 1: Integrated histo-molecular diagnostic of HGG

Glioma patients suffer a wide range of symptoms. Focal or generalized symptoms vary depending on the size and location of the tumor, as well as the degree of peritumoral edema. The most prevalent symptoms include seizures, cognitive deficits, drowsiness, dysphagia, headache, confusion, aphasia, motor deficits, fatigue, and dyspnea [21].

The objective of imaging tests of brain tumors is to detect lesions, locate them, define their extension, and characterize them. The gold standard is magnetic resonance imaging (MRI) with contrast [22]. Advanced MRI techniques include perfusion-weighted imaging (PWI), diffusion-weighted imaging (DWI), and proton magnetic resonance spectroscopy (MRS) [23]. DWI and PWI provide optimal diagnostic performance in differentiating pseudoprogression from true tumor progression; neither technique have proven superiority [24]. Nuclear Medicine Imaging include PET 18 F-FDG, 11C-Met, FET, and FDOPA. They can provide additional support to establish the diagnosis of HGG [25].

#### Recommendations

- Glioma classification should follow the 5th edition of the WHO classification (2021) and complemented by cIMPACT-NOW updates (IV, B).
- Immunohistochemistry for mutant IDH1 R132H protein and nuclear expression of ATRX should be performed routinely in the diagnostic workup for diffuse gliomas (IV, B).
- 1p/19q codeletion status should be determined in IDH-mutant gliomas with retained nuclear expression of ATRX (II, B).
-  + 7/− 0 signature, EGFR amplification, and TERT promoter mutation should be tested in all IDH-WT diffuse gliomas lacking microvascular proliferation and necrosis as histological features of WHO grade 4 to allow for a diagnosis of grade 4 IDH WT glioblastoma (IV, B).
- Assessment of H3 K27M status should be performed in diffuse gliomas involving the midline (IV, B).
- In patients with a suspected GB, T2-weighted, FLAIR, and pre- and post-gadolinium contrast enhanced T1-weighted MRI imaging are recommended. The addition of DWI and PWI can aid in the assessment of suspected GB for the purposes of distinguishing GB from other tumor types (II).
- MRS and nuclear medicine imaging can be used to provide additional support for the diagnosis of GB (III).

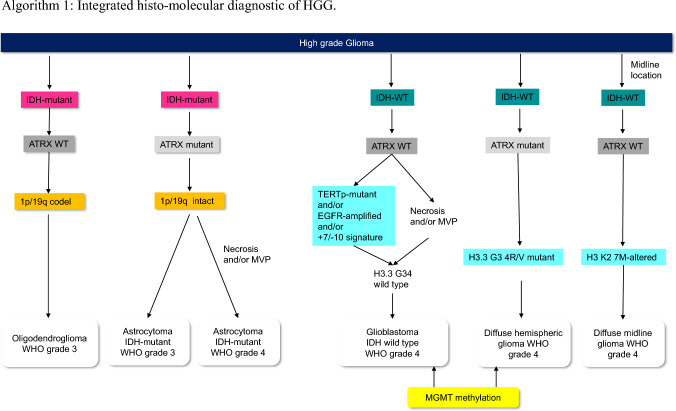


### First line treatment for HGG

Despite the growth of knowledge concerning the molecular biology of gliomas in recent years, GB remains a tumor with a dismal prognosis, with an overall survival (OS) rate of approximately 15–20 months [26, 27] and 5-year survival of < 10% [28]. The first line of care consists of a multimodal treatment approach with surgical resection, RT, and CTX. Unfortunately, none of these strategies are curative, and clinical trials are the preferred option when feasible. The extent of resection has been recently validated as a prognostic marker [29]. After maximal safe resection, the standard therapy (Stupp protocol) remains RT with concurrent temozolomide (TMZ) 75 mg/m^2^/day for 6 weeks and maintenance TMZ (150–200 mg/m^2^/day × 5 days for 6 cycles) [26] (I, A). Extending the length of adjuvant TMZ beyond 6 cycles has not demonstrated survival benefit [30]. After concomitant RT-TMZ, adding tumor-treating fields (TTF) during the adjuvant TMZ phase prolonged OS by a median of 4.9 months in one open-label randomized study [31]. Other strategies have been explored in newly diagnosed glioblastoma such as dose-dense TMZ or the addition of bevacizumab in phase III trials, however none proved a survival benefit [27, 32, 33]. Nevertheless, the combination of temozolomide and lomustine in patients with *MGMT* promoter-methylated glioblastoma extended OS from 31.4 months with TMZ alone to 48.1 months with lomustine-TMZ in a recent, small, phase III trial [34]. This study nevertheless did not report superior progression free survival (PFS) for the combination, the survival curves separated after 2–3 years and the univariate analysis showed a small effect. In light of the above mentioned findings and given that hematologic toxicity was higher for the combination, this strategy is not currently used in our country.

Treatment with immune checkpoint blockade has shown improved survival in murine glioma models. However, data from two phase III studies in newly diagnosed glioma patients with the anti PD-L1 nivolumab did not meet their primary endpoint of OS in the final analysis [35, 36]. For MGMT unmethylated GB, said studies compared nivolumab concurrent with RT follow by nivolumab until disease progression or unacceptable toxicity *versus* Stupp protocol [35]. While for newly diagnosed patients with MGMT methylated or indeterminate GB the standard treatment was compared to the same scheme plus nivolumab [36]. One interesting feature of the first trial was the baseline PD-L1 expression in tumor tissue: < 1% in > 55% in the RT-TMZ arm and > 62% in the RT-nivolumab-treated group. While debate still rages regarding the role and predictive value of this biomarker, as well as optimal threshold, such a high level of lack of expression of a key mechanistic molecule is worrisome.

Surgical intervention provides the greatest survival benefit, while patient age > 70 years old is the worst prognostic factor. Annual GB incidence rates will continue to increase by almost 50% in the upcoming 30 years as the population ages and this trend is likely to continue and increase. Nevertheless, no patients aged ≥ 70 years were included in the initial trial that tested standard treatment [28]. The phase III NOA-08 trial [37] established the non-inferiority of TMZ with respect to standard radiotherapy in a population aged ≥ 65 years (Event free survival of 3.3 months vs 4.7 months; HR 1.15; 95% CI 0.92–1.43; *p* non-inferiority = 0.043). The novelty of this trial lies in the predictive role of MGMT promoter methylation. In subjects receiving TMZ, methylation exhibited longer PFS (8.4 months vs 4.6), while in the unmethylated population, RT appeared superior to TMZ for the PFS endpoint. The EORTC 26062-22061/NCIC CTG Intergroup trial randomized participants to concomitant treatment with short-course hypofractionated RT + TMZ versus short-course hypofractionated RT alone (40 Gy in 15 fractions) in 562 patients > 65 years of age [38]. The combination of TMZ + RT resulted in better OS (9.3 months vs. 7.6 months; HR for death, 0.67; 95% CI 0.56–0.80; *p < *0.001) and PFS (5.3 months vs. 3.9 months; HR for disease progression or death, 0.50; 95% CI 0.41–0.60; *p < *0.001), although MGMT promoter methylation continued to be the main predictor of survival (13.5 months with RT + TMZ and 7.7 months with RT alone (HR for death, 0.53; 95% CI 0.38–0.73; *p < *0.001). However, it is unclear as to whether this scheme is only indicated for the fit elderly patients and as to the best method to assess functionality in the elderly population diagnosed with GB. On the other hand, there are no randomized trials using geriatric assessments in elderly people with GB, in spite of the fact that the frailty index was the prognostic factor that correlated most with survival in elderly patients treated with CT + RT or RT in one small trial [39].

Treatment of grade 3 IDH-mutant astrocytomas has recently been defined based on the results of the CATNON trial [40]. The EORTC 26053 trial (CATNON) randomized patients to radiotherapy alone or with concomitant TMZ and/or with maintenance TMZ (12 cycles) and showed a significant OS improvement with the addition of 12 cycles of maintenance TMZ after radiotherapy (median overall survival 82.3 vs 46.9 months; HR 0.64 [95% CI 0.52–0.79]; *p < *0·0001) and is considered the standard of care for grade 3 IDH-mutant astrocytomas. The role of concomitant TMZ remains uncertain. Molecular analyses of the CATNON trial indicate that only individuals with IDH-mutant tumors derive benefit from maintenance TMZ (OS 116 vs 77 months; 5-year survival 81% vs 62%) [40–42].

As for first-line treatment of WHO grade 3 oligodendrogliomas, two large, randomized clinical trials demonstrated that adding PCV (procarbacine + lomustine + vincristine) to radiotherapy approximately doubled OS (RTOG 9402 7.3 vs 13.2 years; EORTC 26951 9.3 vs 14.2 years), thereby defining the standard of care for this population. The modified CODEL trial will address whether TMZ-based chemoradiotherapy has similar effectiveness as PCV following RT in these cases [43–45].

Grade 4 IDH-mutant astrocytomas are a new glioma entity and there is no standard treatment, because there are not randomized trials in this setting. They may be treated like grade 3 IDH-mutant astrocytomas (RT followed by TMZ × 12 cycles) or grade 4 IDH-WT GB patients (Stupp regimen).

#### Recommendations

- The standard of care for newly diagnosed GB patients is chemoradiotherapy + TMZ, Stupp regimen (I, A).
- Clinicians should consider treatment with TTF (alternating electric field therapy) and TMZ in subjects without suspected progression or pseudoprogression after chemoradiation with TMZ, if available in the center (I, A).
- In elderly or frail patients and/or those with a poor Karnofsky status, monotherapy with TMZ or radiotherapy is suggested, depending on MGMT status (II-B).
- The standard of care in the elderly with a good Karnofsky status newly diagnosed with GB is a short course of RT combined with concomitant and sequential TMZ (I, A).
- The standard of care for patients with newly astrocytoma grade 3 IDH-mutant is radiotherapy followed by 12 cycles of maintenance TMZ (I-A).
- The standard of care for newly diagnosed grade 3 oligodendroglioma is RT + PCV polychemotherapy (PCV) (I, A).

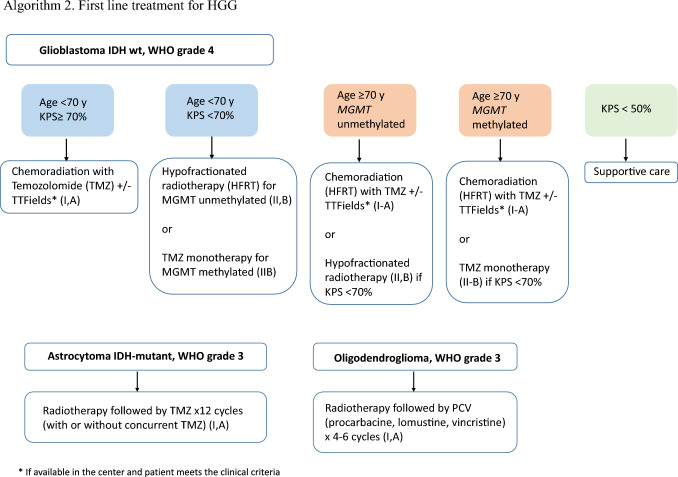


### Management of recurrent disease

There is no standard second-line therapy for recurrent HGG. Treatment should be individualized and based on multidisciplinary tumor board recommendations, depending on previous treatment, time of relapse, patient’s performance status, corticosteroid requirement, and molecular marker profile [46]. Enrolment in a clinical trial (if possible) is the preferred option.

### Surgery

Second surgical resection can be offered to a limited number of cases (II, A). Efficacy studies are based on retrospective cohorts and there is no consensus regarding its benefits for survival and quality of life [47]. Recently, an exploratory retrospective study has revealed increased survival when complete resection of the total contrast enhancement area was performed [48]. A second surgery is considered if the patient maintains good performance status; gross total resection is planned, and the interval since the initial surgery exceeds 6 months, to avoid the risk of pseudo-progression and those patients with fast progression and poor prognosis [49].

### Re-irradiation

The efficacy of re-irradiation remains controverted. There is a paucity of randomized trials demonstrating survival benefits. The only randomized clinical trial exploring bevacizumab + radiotherapy *versus* bevacizumab alone reported improved PFS, but not OS [50]. Nevertheless, there is retrospective evidence for improved outcomes with stereotactic radiosurgery (SRS) and short-course hypofractionated stereotactic RT [51]. There is no standard regarding dose fractionation, regimen, target volume, or stereotactic system. As most recurrences occur in previously irradiated brains, RT can be considered when a long interval has elapsed since the previous RT treatment and if the response to prior RT treatment was good.

### Systemic treatment: chemotherapy options

Lomustine is the most widely accepted standard of care for GB recurrence and HGG recurrence [52, 53]. It has largely been used as the standard control arm in randomized clinical trials. However, it has never been proven superiority over any another agent and has reported a modest 6-month PFS rate of 20%. Other nitrosoureas like carmustine, fotemustine, or PCV have also demonstrated activity in recurrent GB and HGG phase II studies [54, 55]. Re-treatment with TMZ is also a valid option for subjects with a long interval (usually > 4–6 months) between completion of adjuvant TMZ, especially if in cases of MGMT methylated tumors [56, 57]. Alternative TMZ dosing schedules and intense dosing have not shown superiority over standard dosing [58].

### Systemic treatment: antiangiogenics

Phase II clinical trials using bevacizumab in monotherapy and a randomized phase III clinical trial evaluating the combination of bevacizumab + lomustine versus lomustine alone in recurrent GB have only shown improved PFS, but not OS [59]. In light of these results, bevacizumab was not approved for this indication in the European Union, as well as having no financial approval from Spanish regulatory agencies. In clinical practice, the main value of bevacizumab is edema-related symptom relief in symptomatic patients with large tumors. Bevacizumab has been approved by the FDA in United States of America based on objective responses rates of 30% in two, uncontrolled phase II trials and could be a treatment option for patients with brain edema requiring corticosteroids. Regorafenib has been approved in Italy, since the randomized phase II REGOMA study demonstrated an OS benefit in recurrent GB when compared to lomustine [60]; more data is awaited from the AGILE trial to confirm its efficacy.

### Systemic treatment: targeted therapy

Currently, no targeted therapy has been approved by the Spanish regulatory agencies for recurrent GB. However, GB patients harboring a BRAF V600E mutation (approximately 6% of the entire GB population and 50% of epithelioid GB histological subtype) might benefit from BRAF inhibitors [61]. The phase II ROAR trial, using the combination of dabrafenib + trametinib at tumor recurrence has demonstrated a 33% objective response in subjects with HGG [62]. For those harboring *NTRK fusions,* preliminary and exploratory data from early basket trials with NTRK inhibitors (larotrectinib and entrectinib) pointed toward a favorable safety profile and potential benefit in terms of response [63, 64].

### Other therapies for recurrent GB

Other treatment options evaluated in phase II and III clinical trials for recurrent GB failed to prolong survival including. These alternatives included TTF treatment [65] and different modalities of immunotherapy, such as immune checkpoint inhibitors [66] and anti-EGFRvIII antigen vaccines [67]. To date, none of these treatment modalities have been approved for recurrent GB, but many new clinical trials exploring new therapeutic opportunities are ongoing.

#### Recommendations

- The standard of care for patients with recurrent HGG has not yet been established (IV, A).
- Whenever possible, enrollment in a clinical trial is the preferred therapeutic option for the management of recurrent disease (II-B).
- Patients with poor performance status should receive palliative/best supportive care (IV, A).
- A second surgery may be indicated for subjects with good performance status, potential gross total tumor resection and ≥ 6 months after the first surgery (IV, B).
- Re-irradiation can be an option in selected cases (IV, B).
- Re-treatment with TMZ can be considered for patients with MGMT methylated tumors and long interval of since the last TMZ doses (II, B).
- Lomustine is the most widely accepted standard for tumor recurrence. Other nitrosoureas have also shown some efficacy in this setting (II, B)
- Bevacizumab has not been approved for recurrent GB in our country, but could be an option in some cases with edema and mass effect (II, B).
- Targeted therapies have not been approved for GB recurrence in our country; nevertheless, we recommend screening for BRAF mutations and NTRK fusions. BRAF inhibitors + MEK inhibitors could be a treatment option in cases of brain tumors harboring the BRAF V600E mutation (II, B), as well as Larotrectinib in patients with brain tumors and NTRK fusions (II, B).
- No immunotherapy has demonstrated efficacy in recurrent GB.

### Follow-up, long-term implications, and survivorship

#### Follow-up

Regular neurological and radiological evaluations are essential in the follow-up of HGG patients. The Neurologic Assessment in Neuro-Oncology (NANO) scale is a useful tool to assess neurological function in clinical trials and also in daily practice [68].

Outside of clinical trials, the first follow-up MRI should be performed approximately one month after completing RT and every three months thereafter unless otherwise clinically indicated. Patients should be scanned on the same MRI equipment during follow-up examinations or at least with the same field strength, to ensure minimal variability.

The Response Assessment in Neuro-Oncology Working Group (RANO) criteria comprise the recommended criteria for radiological assessment of HGG. RANO takes into account signal change on T2/FLAIR sequences and the contrast-enhancing component of the tumor, in addition to clinical data and corticosteroid therapy status.

RANO specifically addressed the issue of so-called pseudoprogression (increased contrast enhancement on imaging 4–12 weeks after the end of RT and concomitant TMZ that may possibly be due to a reactive process and no actual tumor progression). RANO criteria stipulate that, within the first 12 weeks after completion of RT, tumor progression can only be established if most of the new enhancement occurs outside the field of radiation or if histologic confirmation of progression is obtained [69]. There is some evidence that pseudoprogression is more likely to occur in MGMT-methylated tumors [70].

### Long term implications and survivorship

Clinicians must bear in mind that patients, families, and caregivers should not only be warned about diagnosis and treatment but, also pay attention to repeated complications that patients with glioblastoma commonly have to deal with, especially before patient cognitive impairment sets in.

Supportive care alone is an option for subjects with low Karnofsky performance scores, especially if first-line therapy has already been administered.

General patient management includes interventions for the most common complications, such as brain edema, seizures, thromboembolism, neurocognitive deficits, and end-of life care. Good supportive care is partly responsible for the improvement in survival achieved in patients with glioblastoma. The level of evidence for these questions is low; therefore, most of the recommendations are based mainly on consensus and expert opinions.

### Brain edema

Systemic glucocorticoids are key in symptomatic management of peritumoral edema. Dexamethasone is known to be the preferred steroid for treatment, largely due to its low mineralocorticoid effects, easy administration, and long half-life. The standard doses are usually between 4 and 16 mg/day; the lowest effective dose is recommended [71]. There are few alternatives to steroids, but bevacizumab has an antiedema effect, which can be observed as soon as the first dose and may reduce or obviate the need for steroids [72].

Long-term steroid use is associated with side-effects, such as diabetes, myopathy, and infections, especially pneumocystis pneumonia, among others. Clinicians should be aware of these effects and regularly assess the dose of dexamethasone, tapering doses as soon as possible when not needed to control edema.

### Seizures

Prophylactic use of antiepileptics drugs (AEDs) is not recommended to minimize the risk of seizures in newly diagnosed patients. In the perioperative period, there are limited data to endorse recommending AEDs. Neither valproic acid or levetiracetam appears to increase PFS or OS.

Levetiracetam is the best monotherapy option over older AEDs, due to the lack of interactions, easy dosing, oral and intravenous availability, and fewer adverse effects, which are mostly neurocognitive. Lamotrigine is another monotherapy option, recognized in the latest SNO-EANO guideline for anticonvulsant prophylaxis. Its drawbacks include that it is only available for oral use and the long interval necessary to reach optimal dose [73].

Others AEDs may be necessary to control seizures and newer drugs like lacosamide and brivaracetam can also be prescribed, given that they are active in partial seizures and are available for intravenous use [74].

### Venous thromboembolism

Glioblastoma patients are at high risk for venous thromboembolism (VTE). Anticoagulation remains infrautilized, owing to concerns of potential intracranial bleeding. Anticoagulation can be used safely, and low molecular weight heparins (LMWH) are the treatment of choice for venous thromboembolism. Little evidence is currently available regarding efficacy and safety to recommend direct oral anticoagulants (DOACs) [75]. A risk benefit assessment is required for the use of anticoagulation in patients with asymptomatic bleeding on MRI.

Primary thromboprophylaxis with LMWH should be considered in patients hospitalized for a medical complication. Moreover, in surgical scenarios, LMWH should be initiated within 24 h after procedure. Routine primary thromboprophylaxis in the ambulatory setting is not advised [76].

### Neurocognitive impairment

Neurocognitive impairment is a frequent and disabling complication. There are many causal factors, including surgery, disease progression, radiotherapy, AEDs, and brain edema. It is crucial that they be identified, inasmuch as some treatment may be helpful to deal with the impairment; for example, AED-related impairment can be partially overcome by dose adjustments or replacement for another drug.

As for RT treatment, there are no current data indicating a possible benefit or less toxicity from proton therapy over conventional RT.

There is no evidence for pharmacological intervention with drugs used in neurodegenerative dementia such as donepezil or memantine.

In some selected patients, cognitive rehabilitation may be indicated [76].

### End-of-life management

The complications related to clinical decline are multiple and include: agitation, behavioral changes, nutritional and mobility problems, airway secretions etc. [77] Planning for end-of-life is a way to decrease discomfort and psychological distress for patients and their families. Specialized palliative care teams for symptom management and end-life care are recommended [78, 79].

### Final recommendations

**Table:** 

Phrase	Recommendation Grade	Evidence Level
Diagnosis		
Glioma classification should follow the 5th edition of WHO Classification (2021) and complemented by cIMPACT-NOW updates	IV	B
Immunohistochemistry for mutant IDH1 R132H protein and nuclear expression of ATRX should be performed routinely in the diagnostic workup of diffuse gliomas	IV	B
1p/19q codeletion status should be determined in IDH-mutant gliomas with retained nuclear ATRX expression (ATRX wild-type)	II	B
+ 7/−10 signature, EGFR amplification, and TERT promoter mutation should be tested in all IDH-WT diffuse gliomas lacking microvascular proliferation and necrosis as histological features of WHO grade 4 to diagnosis of molecular grade 4 IDH-WT glioblastoma	IV	B
Assessment of H3 K27M status should be done in diffuse gliomas involving the midline	IV	B
In patients with a suspected HGG, a MRI with T2-weighted, FLAIR and pre- and post-gadolinium contrast enhanced T1-weighted imaging is recommended. The addition of DWI and PWI can aid in assessing suspected HGG for the purposes of distinguishing HGG from other processes	II	
MRS and nuclear medicine imaging can be used to provide additional support for a GB diagnosis	III	
First-line treatment		
The standard of care for individuals with newly diagnosed GB is chemoradiotherapy with TMZ, Stupp regimen	I	A
Clinicians should consider treatment with TTF (alternating electric field therapy) and TMZ for patients without suspicion of progression or pseudoprogression following chemoradiation with TMZ (if available in the center)	I	A
In elderly, frail, and/or with patients with poor Karnofsky status, monotherapy with TMZ or RT is suggested depending on MGMT status	II	B
The standard of care for the elderly population with good Karnofsky status and newly diagnosed GB is a short course of RT combined with concomitant and sequential TMZ	I	A
The standard of care for patients with newly diagnosed grade 3 astrocytoma IDH-mutant is RT followed by 12 cycles of maintenance TMZ	I	A
The standard of care for newly diagnosed grade 3 oligodendroglioma is RT followed by PCV polychemotherapy (PCV)	I	A
Recurrent disease		
The standard of care for patients with HGG recurrence has yet to be established	IV	A
Enrollment in a clinical trial, whenever possible, is preferred for the management of recurrent disease	II	B
Patients with poor Karnofsky status should receive palliative/best supportive care	IV	A
Second surgery could be indicated in patients with good performance status, potential gross total tumor resection and > 6 months after the first surgery	IV	B
Re-irradiation may be an option in selected patients	IV	B
Re-treatment with TMZ can be considered in patients with MGMT methylated tumors and a long interval since the prior TMZ doses	II	B
Lomustine is the most widely accepted standard treatment for tumor recurrence. Other nitrosoureas have also demonstrated some efficacy in this setting	II	B
Bevacizumab is not approved for GB recurrence in our country, but it could be an option in some cases with edema and mass effect	II	B
Targeted therapies have not been approved for GB recurrence in our country; nevertheless, we recommend screening for BRAF mutations and NTRK fusions. BRAF inhibitors + MEK inhibitors could be a treatment option for patients with brain tumors harboring the BRAF V600E mutation, as well as larotrectinib in patients with brain tumors and NTRK fusions	II	B

## Data Availability

Not applicable.
